# Black-White Disparities in Overweight and Obesity Trends by Educational Attainment in the United States, 1997–2008

**DOI:** 10.1155/2013/140743

**Published:** 2013-04-10

**Authors:** Chandra L. Jackson, Moyses Szklo, Hsin-Chieh Yeh, Nae-Yuh Wang, Rosemary Dray-Spira, Roland Thorpe, Frederick L. Brancati

**Affiliations:** ^1^Department of Nutrition, Harvard School of Public Health, Boston, MA 02115, USA; ^2^Department of Epidemiology, Johns Hopkins Bloomberg School of Public Health, Baltimore, MD 21205, USA; ^3^Department of Medicine, Johns Hopkins University School of Medicine, Baltimore, MD 21205, USA; ^4^INSERM, U1018, CESP, Occupational and Social Determinants of Health, 75014 Villejuif, France; ^5^Université Paris XI, Villejuif, France; ^6^Université Versailles Saint-Quentin, Versailles, France; ^7^Department of Health Policy and Management, Baltimore, MD, USA

## Abstract

*Background*. Few studies have examined racial and educational disparities in recent population-based trends. *Methods*. We analyzed data of a nationally representative sample of 174,228 US-born adults in the National Health Interview Survey from 1997 to 2008. We determined mean BMI trends by educational attainment and race and black-white prevalence ratios (PRs) for overweight/obesity (BMI > 25 kg/m^2^) using adjusted Poisson regression with robust variance. *Results*. From 1997 to 2008, BMI increased by ≥1 kg/m^2^ in all race-sex groups, and appeared to increase faster among whites. Blacks with greater than a high school education (GHSE) had a consistently higher BMI over time than whites in both women (28.3 ± 0.14 to 29.7 ± 0.18 kg/m^2^ versus 25.8 ± 0.58 to 26.5 ± 0.08 kg/m^2^) and men (28.1 ± 0.17 kg/m^2^ to 29.0 ± 0.20 versus 27.1 ± 0.04 kg/m^2^ to 28.1 ± 0.06 kg/m^2^). For participants of all educational attainment levels, age-adjusted overweight/obesity was greater by 44% (95% CI: 1.42–1.46) in black versus white women and 2% (1.01–1.04) in men. Among those with GHSE, overweight/obesity prevalence was greater (PR: 1.52; 1.49–1.55) in black versus white women, but greater (1.07; 1.05–1.09) in men. *Conclusions*. BMI increased steadily in all race-sex and education groups from 1997 to 2008, and blacks (particularly women) had a consistently higher BMI than their white counterparts. Overweight/obesity trends and racial disparities were more prominent among individuals with higher education levels, compared to their counterparts with lower education levels.

## 1. Introduction

The prevalence of overweight and obesity in the United States has increased at an alarming rate over the past several decades [1, 2], and large disparities between racial and socioeconomic groups have been documented [3]. Different levels of education, suggested as the single most important social influence on health [4], likely contribute to these obesity disparities [5–7], and explanations for the positive association between educational attainment and health are well established [8]. For instance, gradients in health by educational attainment have been long recognized with greater years of education generally associated with healthier behaviors (e.g., no smoking, physical activity, drinking in moderation) as well as access to resources that lead to greater perceived health and physical functioning in addition to lower levels of morbidity and mortality compared to individuals with less years of education [8, 9]. Years of education also appears to be monotonically and linearly associated with cognitive development that my influence health-reasoning ability leading to adoption of prevention strategies that protect health [10, 11]. Associations between educational attainment and overweight/obesity by race and sex, however, remain complex and difficult to understand and mitigate.

Acknowledging that few studies have explored race-specific trends in overweight/obesity according to levels of educational attainment over time [2, 5], we identified a study with a nationally representative US sample that reported noteworthy differences between men and women as well as across racial/ethnic groups [5]. While obesity prevalence increased in all race-sex groups from 1971 to 2000, white women had a clear inverse association between obesity and educational attainment over time, and white men in the low socioeconomic status (SES) group experienced a decrease in obesity from 1999 to 2002. In black women, the association between obesity prevalence and education switched from inverse to the medium-SES group having the highest prevalence by 1999, and obesity increased at a much faster pace among low-SES black men in comparison to their other SES groups. The majority of prior studies have had limited power to robustly investigate racial trends, and have included few black participants [1, 5, 12]. Some studies have relied on nonrepresentative samples, focused on obesity (excluding overweight) or included only one racial/ethnic group [5, 6, 13–16].

To gain a better understanding of current temporal trends related to the influence of educational attainment on overweight/obesity disparities while addressing important gaps in the literature, we used a considerably large, nationally representative sample of the noninstitutionalized US black and white population. We hypothesized that (1) the prevalence of overweight and obesity will have reached a peak among blacks over time, with whites steadily catching up and (2) the racial disparity in overweight/obesity will be wider in groups with higher compared to lower educational attainment, especially among women.

## 2. Methods

### 2.1. The National Health Interview Survey (NHIS)

We analyzed data from NHIS—a series of cross-sectional, nationally representative surveys which used a three-stage stratified cluster probability sampling design to conduct in-person interviews in samples of noninstitutionalized US civilian households. A complete description of NHIS procedures is available elsewhere [17]. In short, each week (on a continuous basis throughout the calendar year), a probability sample of households was interviewed by trained personnel from the US Bureau of the Census to obtain information about health and other characteristics of each member of the sampled household. The interviews were conducted using computer-assisted personal interviewing (CAPI). Information collected for all family members included household composition and sociodemographic characteristics, as well as indicators of health status, activity limitations, injuries, health insurance coverage, and access to and utilization of health care services. From each sampled family, one adult and one child (not included in this analysis) were randomly selected to provide more extensive health-related information. The 12-year average survey response rate among sampled adults was 71.8% (range: 62.6–80.4%). Our study was approved by the Institutional Review Board's Committee on Human Research of the Johns Hopkins Bloomberg School of Public Health.

### 2.2. Study Participants

**Figure 1:**
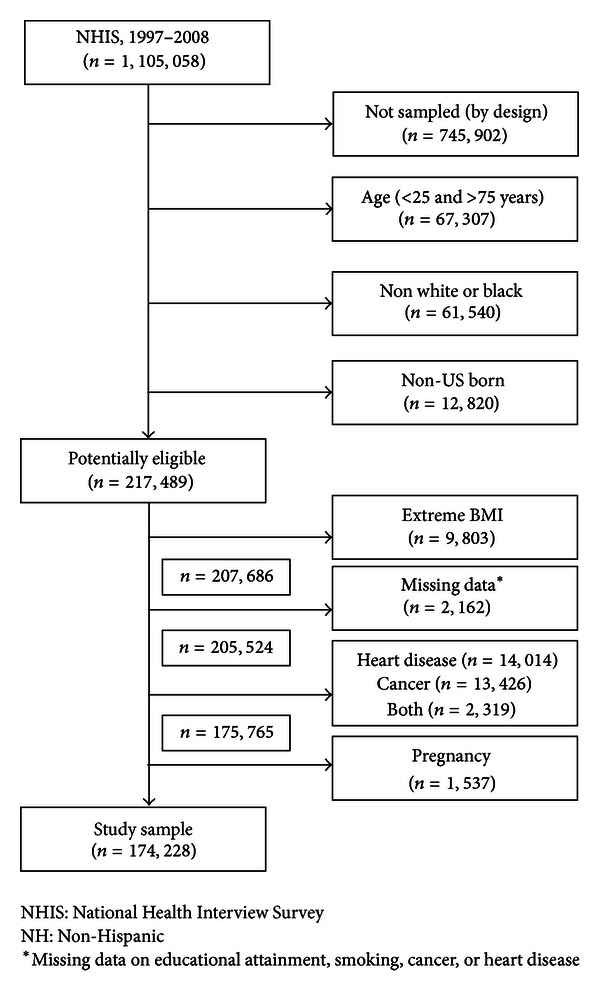
Study flow diagram.

Participants included self-reported non-hispanic white or non-hispanic black (henceforth, white and black) adults aged 25 through 75 years. Participants were excluded if they (1) were born outside the US; (2) reported having a history of cancer and/or heart disease; (3) were pregnant; (4) had missing data on height, weight, educational attainment, or smoking status; or (5) had an extreme body mass index (BMI)—that is, either <15 or >55 kg/m^2^. Our final sample comprised of 174,228 adults (Figure 1). 

### 2.3. Measures

#### 2.3.1. Body Mass Index

Self-reported height and weight were used to calculate BMI (kg/m^2^). Obesity was defined as BMI ≥30 kg/m^2^, overweight as 25.0–29.9 kg/m^2^, normal weight as 18.5–24.9 kg/m^2^, and underweight as BMI <18.5 kg/m^2^. 

#### 2.3.2. Educational Attainment

Educational attainment was categorized as less than high school (<HS) (no high school diploma), high school (HS) (high school or general equivalency diploma), and greater than high school (>HS) (any education beyond high school).

#### 2.3.3. Health Behaviors and Other Variables

Smoking status was categorized as “ever” or “never.” Lifetime alcohol drinking status was assessed and categorized as either “ever” or “never.” Leisure-time physical activity was categorized as none, low, or high based on the participant's answer to the following questions: (1) “How often do you do light or moderate leisure-time physical activities for at least 10 minutes that cause only light sweating or a slight to moderate increase in breathing or heart rate?” and (2) “How often do you do vigorous physical activities for at least 10 minutes that cause heavy sweating or a large increase in breathing or heart rate?” Individuals who answered “never” or “unable to do this type activity” were classified as “none.” Those engaging in at least some level of activity and providing a specific number of activity bouts were dichotomized at the midpoint of these bouts into “low” or “high.” Marital status was categorized as married/living with partner, divorced/separated/widowed, or never married, and regions of the country as South, Midwest, Northeast, and West. 

### 2.4. Statistical Analysis

We used 12 years (1997–2008) of NHIS data merged by the Integrated Health Interview Series [18], a federal effort to create consistent codes and documentation based on public-use data files of the NHIS. For all analyses, sampling weights that account for the unequal probabilities of selection resulting from the sample design, nonresponse, and oversampling of certain subgroups were used. Standard errors or variance estimations were calculated using Taylor series linearization [19]. The STATA “subpop” command was used for correct variance estimation of estimates, and different sampling designs in 1997 to 2005 versus 2006 to 2008 were accounted for by the Integrated Health Interview Series. Modeling assumptions were evaluated where appropriate, and a two-sided *P* value < 0.05 was considered statistically significant. STATA statistical software version 12 (STATA Corporation, College Station, Texas, USA, 2007) was used for all analyses.

Continuous variables were expressed as means ± standard errors (SE), whereas categorical variables were presented as absolute values with corresponding percentages. To test for differences in prespecified sociodemographic, clinical, and behavioral characteristics between whites and blacks and by obesity status, we used the Rao-Scott second-order corrected Pearson statistic [20].

Poisson regression models were used to estimate prevalence ratios and corresponding 95% confidence intervals adjusted for age (in 4 categories: 25–34, 35–49, 50–64, and 65–75 years), marital status, smoking status, alcohol consumption, leisure-time physical activity level, and region of the country [21]. To obtain prevalence ratios for the entire sample, we pooled survey years from three time periods (1997–2000, 2001–2004, and 2005–2008) based on the assumption that the black-white differences in mean BMI remained largely proportional and without crossovers between races by educational level within these study periods. Whites were used as the reference categories for the black-white comparisons. 

Differences in linear trends in mean BMI from 1997 to 2008 between blacks and whites within each educational attainment category were formally tested (at the *α* = 0.05 level) using sex-specific multivariable-adjusted linear regression models where survey year was treated as a dummy variable.

## 3. Results

### 3.1. Characteristics of the Study Population

Sociodemographic, clinical, and behavioral characteristics of the final sample of 174,228 NHIS study participants are shown by race and educational attainment in Table 1. The mean age was 45.8 ± 0.05 years (SE), 51% were men, and 87% were non-hispanic white. Forty-six percent of all participants reported ever smoking in their lifetime, 32% never consumed alcohol, and 34% never engaged or were unable to engage in leisure-time physical activity. Participants' mean BMI was 27.8 ± 0.02 kg/m^2^ for men and 26.8 ± 0.03 kg/m^2^ for women; 26% were obese, 24% reported a diagnosis of hypertension, 6% reported having diabetes, and 10% reported their general health status as either fair or poor.

Blacks were slightly younger than whites, less likely to be married, and more likely to reside in the Southern region of the United States. Blacks were more likely to have less than a high school education, to be obese, to report having hypertension, to report never consuming alcohol, and to report having no leisure-time physical activity. All participant characteristics had less than 10% missing values. We compared participants with complete data versus their counterparts with missing data and found no significant differences in age, sex, race, health status, poverty status, or household size.

### 3.2. Black-White Disparities in Overweight/Obesity Trends by Educational Attainment

**Figure 2:**
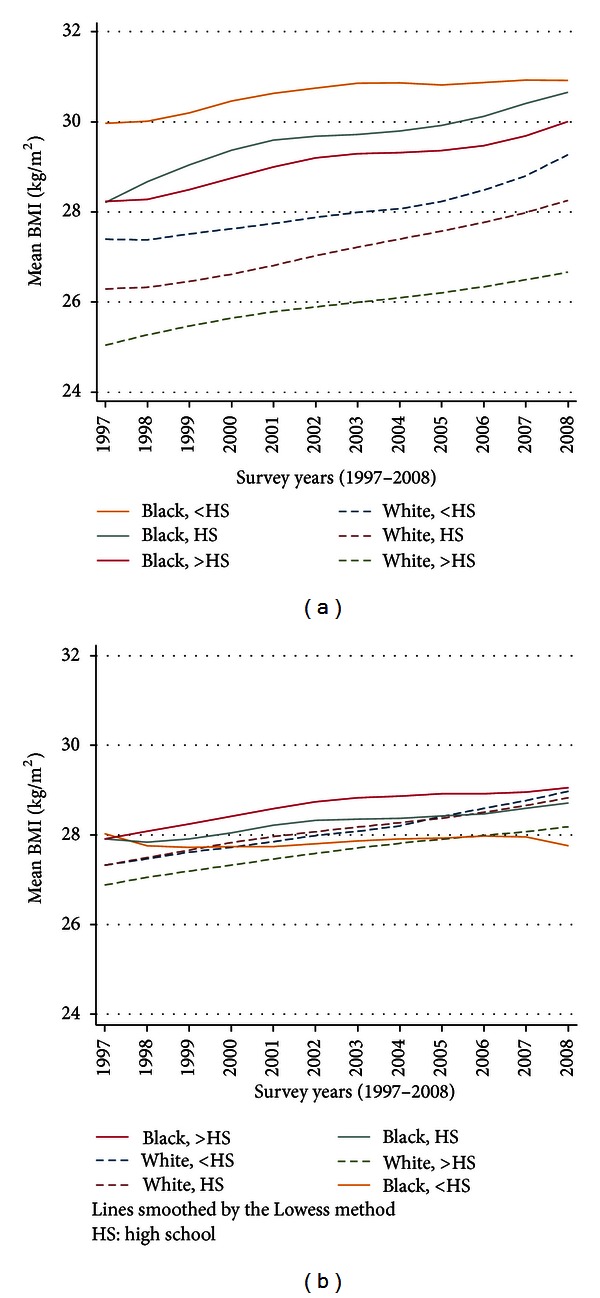
Smoothed trends in mean body mass index among (a) women and (b) men.

From 1997 to 2008, BMI increased by at least 1 kg/m^2^ in all race-sex-education groups (except black men with less than high school education), and mean BMI appeared to increase at a faster pace among whites compared to blacks (Figure 2). Black women had higher mean BMIs compared with white women across the entire study period and across levels of educational attainment, although the greatest racial disparity occurred in women with more than a high school education. In contrast, mean BMI, in black and white men were similar, except among men with more than a high school education, where black men had higher BMIs. Although white women remained the leanest group throughout the study period, their mean BMI exceeded 26 kg/m^2^ by the end of the study period. Among those with greater than a high school education, blacks had a consistently higher BMI over time than whites in both women (28.3 ± 0.14 to 29.7 ± 0.18 kg/m^2^ versus 25.8 ± 0.58 to 26.5 ± 0.08 kg/m^2^) and men (28.1 ± 0.17 kg/m^2^ to 29.0 ± 0.20 versus 27.1 ± 0.04 kg/m^2^ to 28.1 ± 0.06 kg/m^2^). 

 While mean BMIs were different by race (especially among women) the unadjusted slope of BMI increase was significantly different for men (*P* for interaction <0.001) and women (*P* for interaction <0.001) (see Table 2). After adjustment for age, marital status, smoking status, leisure-time physical activity, alcohol consumption, poor income, region of country and self-reported general health status, the slope differences remained significant for men but not for women (*P* for interaction = 0.44). Black men with less than a high school education had stable mean BMIs over time compared to their white counterparts whose BMIs increased over the study period (*P* for interaction = 0.02). Black women had substantially higher BMIs than white women, but the rate of BMI increase did not differ between races with educational attainment combined (*P* for interaction = 0.02) or for any specific level of education. 

### 3.3. Overweight/Obesity Prevalence by Race, Sex, and Educational Attainment

**Figure 3:**
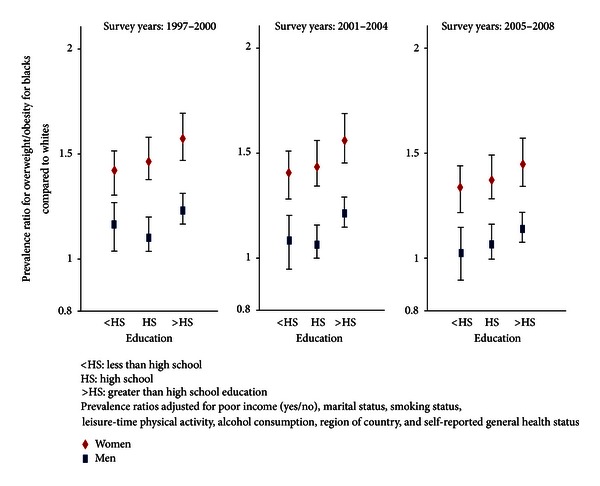
Adjusted prevalence ratios on a log scale for overweight/obesity for blacks compared to whites by sex and educational attainment in 1997 to 2000, 2001 to 2004, and 2005 to 2008.

Figure 3 shows that the black-white overweight/obesity disparity was greatest for women and at education levels greater than high school, which persisted over the study period. For participants of all levels of educational attainment, age-adjusted overweight/obesity was greater by 44% (95% CI: 1.42–1.46) in black versus white women and 2% (95% CI: 1.01–1.04) in black versus white men. Among those with more than a high school education, prevalence of overweight/obesity was much higher (PR 1.52; 95% CI: 1.49–1.55) in black versus white women, but only slightly higher (PR 1.07; 95% CI: 1.05–1.09) in black versus white men. The disparity in overweight/obesity among blacks and whites appears highest among those with more than a high school education for both men and women. This disparity decreased over time as the prevalence of overweight/obesity appears to increase more rapidly for whites compared to blacks. 

## 4. Discussion

 Our analysis of overweight/obesity prevalence trends by sex, race, and educational attainment among US-born Non-Hispanic black and white adults showed that BMI has increased steadily from 1997 to 2008 in all race-sex and education groups, with the exception of black men with less than a high school education, whose prevalence of obesity appeared steady. The racial disparity in overweight/obesity prevalence remained largely proportional over time for each respective education group among women, but the disparity differed by level of educational attainment among men. As a result of the rate of BMI increase being lowest among black men with less than a high school education, there is currently little difference in BMI between black and white men. Blacks (especially women) had a consistently higher BMI than their white counterparts. Although white women remained the leanest group throughout the study period, their mean BMI was above 26 kg/m^2^ at the end of the study. 

 The Coronary Artery Risk Development in Young Adults (CARDIA) study included participants (5,115 black and white men and women) with ages that ranged from 18 to 30 years and found an inverse, cross-sectional association of education with obesity among white women, a positive association among black men, and no significant relationship among both white men and black women [13]. CARDIA participants with limited age ranges had BMI measurements taken once in the late 1980s and were recruited from 4 urban areas of the USA. Nonetheless, this population may not be nationally representative. Another study without trend data found that the largest racial/ethnic disparity in obesity was between US-born black and white women [14]. However, this study also found that high education attenuated the black-white disparity among women and increased the disparities in men, which was in contrast to our study findings [14]. They used data collected from 1988 to 1994 and employed a concentration index to assess socioeconomic inequality in the distribution of obesity.

Using NHANES data pooled from 1999 to 2000, overall, persons in the United States with less than a high school education had a higher prevalence of obesity than their counterparts with more education, with the exception of black women with less than a high school education who had the lowest obesity prevalence compared to those with a higher level educational attainment [14]. In contrast, our study found that the highest overweight/obesity prevalence rates were observed in black women with less than a high school education. 

We identified two studies that investigated time trends in racial differences in obesity prevalence by SES [2, 5]. As previously mentioned, Zhang and Wang investigated trends using the National Health and Nutrition Examination Survey (NHANES) data and found that obesity increased in a complex manner among all SES groups by race/ethnicity and sex from 1971 to 2000 [5]. Between 1976 and 2002, obesity in high- and medium-SES groups of black women increased at a higher rate than low-SES groups. Obesity in the low-SES group of black men increased at a pace faster than other SES groups while the prevalence decreased for white men in the low-SES group between 1988 and 2002.

Another study investigated trends in obesity over time using NHANES data from 1999 to 2004 and found that obesity increased in adults at all education levels [2]. They also found no significant trend in obesity by education among men, but in women a college education was related to a lower prevalence of overweight/obese in comparison to those with less education. In our study, the overweight/obesity disparities increased as educational level increased among blacks and whites for both men and women. The racial difference in overweight/obesity for men was significant only in those with greater than a high school education. These studies had limited sample sizes (especially for black men and women) as well as survey years that only slightly overlapped with our study period. Results may have also differed because NHANES has measured heights and weights to calculate BMI while NHIS relies on self-reported data. Results based on self-reported data from the Behavioral Risk Factor Surveillance System (BRFSS), for instance, showed a clearer linear relation between obesity and education in all race-sex groups compared to NHANES [22]. Similar to our findings, these rates among black women declined with increasing education, although they remained higher than those of white women for all educational levels. 

Although few studies with nationally representative data report small differences in weight and height self-reporting error between blacks and whites of both genders [22–25], the majority of studies conclude that there are no significant differences in weight and height self-reporting error between blacks and whites [26–31]. Yun et al. concluded that the BRFSS (using self-reported data) can correctly identify the population with the highest overweight/obesity burden but did not appear to accurately rank obesity prevalence across various demographic groups (i.e., race and education) [22]. They found that the prevalence of obesity based on self-report was approximately twenty percentage points lower than measured data for black women with more than a high school education [22], which was substantially greater than for other race-sex groups. The discrepancy between NHANES and BRFSS data, therefore, suggests that a difference in self-reported versus measured height and weight data in determining overweight and/or obesity prevalence may be most pronounced among black women. However, the BRFSS collected self-reported height and weight data using telephone surveys, a different modality with stronger associated biases than with in-person interviews used in NHIS [32]. Nonetheless, if these results are accurate, our findings would merely be conservative estimates of overweight/obesity prevalence for black women (in particular). This subject should be explored further in future studies. 

Suggested mechanisms for the generally inverse education-overweight/obesity association include differences in healthy lifestyles and social-psychological resources, in addition to work and economic conditions [8]. A higher educational level has also been shown to encourage health information seeking and comprehension [33] and may act as a builder of social capital [4] and increased personal control of health [8]. Through economic and social advancement, individuals with more education are likely to have, achieve, or maintain high social status or occupations with more earning potential, prestige, and control over decision-making [4, 8]. Educational attainment may specifically influence racial/ethnic disparities in overweight/obesity trends as it has been shown to shape an individual's SES and access to resources (e.g., grocery stores with fresh produce) and opportunities (e.g., sidewalks for physical activity) that afford or compromise healthy lifestyles and weight status through historical and pervasive differential acquisition of occupations, incomes, and neighborhood choices [7, 34]. Supportive relationships and social support, self-image related to desired weight, knowledge of nutrition, and access to tools for weight control are also likely contributors to observed disparities [7, 34]. 

Although it has been widely accepted that low-SES US groups are at increased risk of obesity [35], complexities still exist regarding relationships of sex, ethnicity, and SES with obesity [36, 37]. Less-educated persons in the USA have been consistently shown to have a higher prevalence of obesity than their more educated counterparts, with the exception of black women [6]. Black women with less than a high school education have been shown to have the lowest obesity prevalence compared to black women with higher educational levels.

There are several limitations of our study that deserve to be mentioned. First, our data are based on self-report, and thus differential misclassification by race in ascertaining height and weight to estimate BMI is possible. Second, the three educational levels were fairly broad, and it is likely that blacks are closer to the lower cut-off of each education level than whites, which may result in residual confounding. Additionally, even at the same levels, education, as a marker of SES, may not have the same social and health benefits or construct validity across racial/ethnic groups [5, 14]. 

Important to our relationships of interest, we were also unable (like many studies) to assess education quality versus attainment, adjust for finer categories of smoking status, which is associated with lower BMI, or discern cohabiting couples from those who were married or single. Despite these limitations, our study has several strengths. First, the sample size was large, allowing stratification by race and educational attainment. Second, we had a relatively large black population, which affords more robust estimates than those from previous studies as this is the largest sample of the US population. In addition, we used a nationally representative sample of the USA. 

These data help enable planners to develop more effective public health strategies and direct resources to subpopulations with exceptionally high (or increasing) obesity for targeted intervention and in-depth research. As most race-sex-education groups have been affected by the obesity epidemic, a growing consensus of stakeholders agrees that population-based policies and programs emphasizing environmental changes are most likely to be successful in addressing the obesity epidemic. 

This study underscores substantial and complex differences in obesity prevalence by education (especially among women), which have persisted over time. Black women with greater than a high school education had substantially higher mean BMIs than even white women with less than a high school education. Our study suggests that mean BMI appears to be increasing at a faster pace among whites than blacks and racial disparities in overweight/obesity trends and prevalence were more prominent among more educated individuals as compared to their less-educated counterparts. Higher education does not appear protective against the obesity epidemic nor racial/ethnic disparities in overweight/obesity.

## Figures and Tables

**Table 1:** Sociodemographic, health behavior, and clinical characteristics of National Health Interview Survey (NHIS) participants, according to educational attainment and race/ethnicity, 1997–2008 (*N* = 174,228).

	<HS		HS		>HS		Combined education		
	White	Black	White	Black	White	Black	White	Black	Total
Sample size, *N* (%)	11,987 (76)	5,617 (24)	45,174 (85)	11,316 (15)	85,189 (89)	14,945 (11)	142,350 (87)	31,878 (13)	174,228
Age									
Mean, year ± SE	50.9 ± 0.15	50.5 ± 0.27	47.4 ± 0.08	43.9 ± 0.15	44.6 ± 0.07	42.1 ± 0.13	46.0 ± 0.05	44.1 ± 0.11	45.8 ± 0.05
Age group—%									
25–34	18	18	20	27	25	31	23	28	23
35–49	30	30	39	43	42	43	40	41	40
50–64	29	31	28	23	26	21	27	23	27
65–75	23	21	13	7	7	5	10	8	10
Male	53	46	51	49	51	43	51	46	51
Marital status									
Married/living w/partner	59	33	68	41	69	46	68	42	65
Divorced/separated/widowed	28	36	20	27	16	25	18	27	19
Never married	13	31	12	32	15	29	14	31	16
Health behaviors									
Ever smoker (yes)	68	58	57	44	42	34	49	41	46
Alcohol consumer									
Never	46	50	36	48	24	47	30	48	32
Ever	54	50	64	52	76	53	70	52	68
Leisure-time physical activity									
Never/unable	59	67	42	53	24	36	33	47	34
Low	19	17	29	25	38	35	34	29	33
High	22	16	29	22	38	29	34	24	32
Clinical characteristics									
Mean BMI (kg/m^2^), men	27.9 ± 0.09	27.8 ± 0.13	28.0 ± 0.04	28.2 ± 0.09	27.6 ± 0.03	28.7 ± 0.08	27.7 ± 0.02	28.4 ± 0.06	27.8 ± 0.02
Mean BMI (kg/m^2^), women	27.7 ± 0.10	30.6 ± 0.15	27.0 ± 0.05	29.6 ± 0.10	25.9 ± 0.04	29.1 ± 0.09	26.4 ± 0.03	29.5 ± 0.07	26.8 ± 0.03
Overweight/obese (yes)	66	73	65	73	59	72	62	72	63
Obesity (yes)	31	40	28	36	22	35	25	36	26
Health conditions									
Hypertension (yes)	34	48	26	32	19	28	23	33	24
Diabetes (yes)	11	16	6	9	4	8	5	9	6
General health status									
Excellent/very good	39	32	61	50	77	63	69	54	66
Good	34	32	29	33	18	27	23	30	24
Fair/poor	27	36	10	17	5	10	9	17	10
Region of country									
Northeast	15	12	20	13	19	13	19	13	18
Midwest	24	17	30	19	28	20	28	19	27
South	47	67	35	61	33	57	35	60	38
West	14	4	15	7	20	10	18	8	17

Weighted estimates; mean ± SE or (%); SE: standard error; <HS: less than high school, HS: high school, >HS: greater than high school.

**Table 2:** Slope differences for BMI and survey year between Blacks compared to Whites among men and women, overall and by educational attainment, 1997–2008.

	*β* coefficient, unadjusted	95% confidence interval	*P* value	*β* coefficient, adjusted	95% confidence interval	*P* value
Men						
<HS	−0.11049	(−0.20641–−0.01457)	0.02	−0.11840	(−0.21388–−0.02291)	0.02
HS	−0.05580	(−0.11559–0.00399)	0.07	−0.04504	(−0.10294–0.01287)	0.13
>HS	−0.01283	(−0.06770–0.04205)	0.65	−0.01317	(−0.06803–0.04169)	0.64
Combined education	0.00020	(0.00012–0.00027)	<0.001	0.00030	(0.00023–0.00037)	<0.001
Women						
<HS	−0.02087	(−0.13259–0.09085)	0.71	−0.03837	(−0.14755–0.07082)	0.49
HS	−0.00447	(−0.06656–0.05762)	0.89	−0.0004454	(−0.06251–0.06162)	0.99
>HS	0.01973	(−0.03016–0.06961)	0.44	0.00351	(−0.04473–0.05174)	0.89
Combined education	−0.00037	(−0.00044–−0.00029)	<0.001	−0.00003	(−0.00010–0.00004)	0.44

*β*: beta; <HS: less than high school, HS: high school, >HS: greater than high school.

Adjusted model: age (4 categories), marital status, smoking status, leisure-time physical activity, alcohol consumption, poor income, region of country, and self-reported general health status; *P* values represent sex-specific slope differences between Blacks and Whites by education level from linear regression models; combined interaction term race, education and survey year.
